# Dynamic ^18^F-FDopa PET Imaging for Newly Diagnosed Gliomas: Is a Semiquantitative Model Sufficient?

**DOI:** 10.3389/fonc.2021.735257

**Published:** 2021-10-05

**Authors:** Timothée Zaragori, Matthieu Doyen, Fabien Rech, Marie Blonski, Luc Taillandier, Laëtitia Imbert, Antoine Verger

**Affiliations:** ^1^Department of Nuclear Medicine and Nancyclotep Imaging Platform, CHRU-Nancy, Université de Lorraine, Nancy, France; ^2^IADI UMR 1254, INSERM, Université de Lorraine, Nancy, France; ^3^Department of Neurosurgery, CHRU-Nancy, Université de Lorraine, Nancy, France; ^4^Centre de Recherche en Automatique de Nancy CRAN UMR 7039, CNRS, Université de Lorraine, Nancy, France; ^5^Department of Neuro-Oncology, CHRU-Nancy, Université de Lorraine, Nancy, France

**Keywords:** DOPA, PET, compartmental modeling, dynamic analysis, glioma, IDH mutation

## Abstract

**Purpose:**

Dynamic amino acid positron emission tomography (PET) has become essential in neuro-oncology, most notably for its prognostic value in the noninvasive prediction of isocitrate dehydrogenase (IDH) mutations in newly diagnosed gliomas. The 6-[^18^F]fluoro-l-DOPA (^18^F-FDOPA) kinetic model has an underlying complexity, while previous studies have predominantly used a semiquantitative dynamic analysis. Our study addresses whether a semiquantitative analysis can capture all the relevant information contained in time–activity curves for predicting the presence of IDH mutations compared to the more sophisticated graphical and compartmental models.

**Methods:**

Thirty-seven tumour time–activity curves from ^18^F-FDOPA PET dynamic acquisitions of newly diagnosed gliomas (median age = 58.3 years, range = 20.3–79.9 years, 16 women, 16 IDH-wild type) were analyzed with a semiquantitative model based on classical parameters, with (SQ) or without (Ref SQ) a reference region, or on parameters of a fit function (SQ Fit), a graphical Logan model with input function (Logan) or reference region (Ref Logan), and a two-tissue compartmental model previously reported for ^18^F-FDOPA PET imaging of gliomas (2TCM). The overall predictive performance of each model was assessed with an area under the curve (AUC) comparison using multivariate analysis of all the parameters included in the model. Moreover, each extracted parameter was assessed in a univariate analysis by a receiver operating characteristic curve analysis.

**Results:**

The SQ model with an AUC of 0.733 for predicting IDH mutations showed comparable performance to the other models with AUCs of 0.752, 0.814, 0.693, 0.786, and 0.863, respectively corresponding to SQ Fit, Ref SQ, Logan, Ref Logan, and 2TCM (*p* ≥ 0.10 for the pairwise comparisons with other models). In the univariate analysis, the SQ time-to-peak parameter had the best diagnostic performance (75.7% accuracy) compared to all other individual parameters considered.

**Conclusions:**

The SQ model circumvents the complexities of the ^18^F-FDOPA kinetic model and yields similar performance in predicting IDH mutations when compared to the other models, most notably the compartmental model. Our study provides supportive evidence for the routine clinical application of the SQ model for the dynamic analysis of ^18^F-FDOPA PET images in newly diagnosed gliomas.

## Introduction

As an adjunct to magnetic resonance imaging (MRI), amino acid positron emission tomography (PET) imaging provides additional diagnostic and prognostic information in newly diagnosed gliomas (1). Amino acid PET is particularly helpful for gliomas exhibiting no contrast enhancement on MRI (2–4), which can, in some cases, conceal more aggressive gliomas (5). The most commonly used radiolabels, i.e., amino acids *O*-(2-[^18^F]fluoroethyl)-l-tyrosine (^18^F-FET) (6), ^11^C-methionine (^11^C-MET) (7), and 6-[^18^F]fluoro-l-DOPA (^18^F-FDOPA), were able to noninvasively characterize gliomas at the initial diagnosis (8). Dynamic acquisitions are started at the time of tracer injection, to follow the full kinetic path, and are most commonly performed with ^18^F-FET and ^18^F-FDOPA. Dynamic ^11^C-MET acquisitions are rarely performed because of the limitations of the short (20 min) half-life of this radiolabeled amino acid (9).

Dynamic ^18^F-FET and ^18^F-FDOPA acquisitions have recently shown encouraging predictive performances for the noninvasive characterization of the mutation status of isocitrate dehydrogenase (IDH) in newly diagnosed gliomas (6, 8). This is considered to be one of the most important molecular parameters in gliomas according to the 2021 classification of the World Health Organization (WHO) (10). These results were obtained using a semiquantitative model, initially developed for dynamic ^18^F-FET PET imaging, that can be easily transposed to the clinic (11). The model relies on the extraction of two parameters directly from the time–activity curve (TAC) without requiring any modeling of the underlying metabolism. These parameters are the time-to-peak (TTP), which is the time from the beginning of the dynamic acquisition to the maximal TAC value, and the late phase slope of the TAC. This simple model is currently recommended for dynamic ^18^F-FET PET analyses (12) and has been reported to predict IDH mutation with an accuracy of 72% (6). The same dynamic analysis has also been successfully transposed to ^18^F-FDOPA dynamic images, with the time-to-peak parameter demonstrating a promising 74% accuracy for predicting the presence of IDH mutations in newly diagnosed gliomas (8).

The simple dynamic ^18^F-FET PET modeling approach can be applied because, unlike other radiolabeled amino acids, ^18^F-FET is not metabolized by cells (13). Indeed, several studies have shown that ^18^F-FET kinetics are best modeled using a one-tissue reversible compartmental model with reliable fit and stable kinetic parameters (14–16). The ^18^F-FDOPA kinetics in glioma, however, involve a much more complex model that is, to date, only partially characterized (17, 18) because the initial models were developed in the context of assessing Parkinson’s disease (19). The complexities of the ^18^F-FDOPA kinetic model arise from its different peripheral and intracellular metabolic pathways. ^18^F-FDOPA is metabolized into ^18^F-labeled metabolites (METS) and ^18^F-labeled l-3,4-dihydroxy-6-fluoro-3-*O*-methylphenylalanine (OMFD) in the periphery, with OMFD able to bidirectionally cross the blood–brain barrier. In addition, and unlike ^18^F-FET, ^18^F-FDOPA is metabolized *via* the dopaminergic pathway, even though it is unclear whether it is metabolized in the same way by tumor cells. A two-tissue compartmental model, only using one input function, has nevertheless been previously proven useful for the ^18^F-FDOPA imaging of gliomas (17, 18). However, to the best of our knowledge, there are currently no studies that compare the semiquantitative dynamic model to other dynamic models, such as the compartmental ^18^F-FDOPA PET imaging model in gliomas (17, 18).

Our current study assesses whether an ^18^F-FDOPA semiquantitative dynamic analysis indeed captures all the relevant information contained in time–activity curves to predict the presence of IDH mutations compared to the more sophisticated graphical and compartmental models.

## Materials and Methods

### Patients

We retrospectively selected newly diagnosed glioma patients for whom ^18^F-FDOPA PET had been performed as part of the initial tumor characterization, in the Department of Nuclear Medicine at the CHRU of Nancy, between February 2018 and June 2020. The final selection included: i) patients with a neuropathological diagnosis based on the WHO 2016 classification (20) and with a maximum time interval of 150 days between the ^18^F-FDOPA PET and the histological confirmation for diffuse grade II or III gliomas and 60 days for glioblastomas (6, 8); ii) patients with available raw data for *a posteriori* reconstruction; and iii) patients with a visually abnormal ^18^F-FDOPA uptake, i.e., by excluding isometabolic and photopenic gliomas (21, 22). The institutional ethics committee (Comité d’Ethique du CHRU de Nancy—FRANCE) approved the evaluation of retrospective patient data on August 26, 2020. The trial was registered at ClinicalTrials.gov (NCT04469244). This research complied with the principles of the Declaration of Helsinki. Informed consent was obtained from all individuals included in the study.

### ^18^F-FDopa PET Acquisition and Image Reconstruction

Patients were instructed to fast for at least 4 h prior to the examination and were pre-medicated with Carbidopa 1 h prior to the examination to increase tracer uptake in the brain (17). Patients were scanned with a digital Vereos PET/computed tomography (CT) camera (Vereos, Philips, Cleveland, OH, USA). Immediately after recording the CT images (100 kV, 80 mAs), a 30-min 3D list-mode PET acquisition was initiated concomitantly to the bolus injection of 2 MBq of ^18^F-FDOPA per kilogram of body weight. Static PET images were reconstructed from the list-mode data acquired 10–30 min post-injection (12, 23). Dynamic images of all patients were reconstructed using two different temporal sampling protocols depending on the dynamic analyses carried out. For models requiring an input function, we used the recommended temporal sampling protocol from the EANM/SNMMI guidelines, i.e., 12 frames of 5 s, 6 frames of 10 s, 6 frames of 30 s, 5 frames of 60 s, and 4 frames of 300 s (12). This protocol was used because very short frames at the start of the acquisition are needed to capture the large variations of the radiotracer concentration in the blood that occur at the beginning of the acquisition. The temporal sampling protocol consisted of 30 × 60 s frames for models not requiring any input function (24). For models that do not require an input function, the short initial frames only contribute very noisy data points that do not contain any useful information because vascular phase data are not used. Moreover, using a uniform frame duration has the advantage of eliminating one source of noise due to the variations between the time points of the TACs.

Static images were reconstructed using the time-of-flight information and a high-resolution protocol with the Ordered Subset Expectation Maximization (OSEM) 3D algorithm [two iterations, 10 subsets, a deconvolution of the point spread function (PSF), and 256 × 256 × 164 voxels of 1 × 1 × 1 mm^3^], while a protocol with a lower spatial resolution was used to limit the level of noise in dynamic images, i.e., three iterations, 15 subsets, without PSF, and 128 × 128 × 82 voxels of 2 × 2 × 2 mm^3^ (25).

All images were corrected for attenuation using CT, dead time, and random and scattered coincidences during the reconstruction process.

### Segmentation

Healthy brain uptake was initially measured from static images using a merged volume of interest (VOI) consisting of a crescent-shaped region of interest manually positioned on three consecutive slices of the semi-oval center of the unaffected hemisphere to include both white and gray matter. Tumor VOI was segmented semi-automatically from static images using a threshold of 1.6 healthy brain SUV_mean_, as previously recommended (24, 26, 27). The arterial input function VOI was subsequently placed into the internal carotid using initial dynamic frames to identify the early vascular phases (28).

All volumes of interest were segmented using the LifeX software (lifexsoft.org) (29) and were visually inspected by an experienced physician (AV, nuclear physician with more than 10 years of experience) to ensure the quality of the methods applied. Healthy brain was considered as the reference region due to its nonspecific uptake, as required (26).

### Extraction of Time–Activity Curves

For dynamic images reconstructed using the protocol with 30 × 60 s frames, each dynamic frame was first registered to the associated CT image in order to correct for any potential patient movement during the acquisition (30). These transformations, representing the evolution of the patient’s movements over time, were interpolated to the time frames of the other protocol, for models involving an input function. Indeed, the first frames from images reconstructed with models involving an input function are very short and suffer from noise, which makes the registration very challenging.

Blood and brain TACs were extracted by retrieving the mean standard uptake value (SUV_mean_) for each frame in the respective VOIs. Tumor TACs were computed by retrieving the SUV_mean_ for each frame in the volume corresponding to the SUV_peak_ of the tumor VOI on the static image in order to represent the most aggressive part of the tumor (24).

### Input Function Pre-Processing

Since no arterial blood sampling was performed in this study, an image-derived input function was used for analyses that required one. TACs representing the evolution of the arterial blood activity were obtained from internal carotid VOIs and were fitted using linear interpolation to the peak followed by a tri-exponential function after the peak (31). The fitted blood TAC was then corrected for the spill-out effect, the coefficient of which had been estimated as 0.51 (32). In the case of ^18^F-FDOPA, the plasma ^18^F-FDOPA TAC was obtained after correcting for OMFD and other METS generated in the peripheral tissues (19). The plasma ^18^F-FDOPA TAC can be obtained from the blood TAC if the hematocrit level and the proportion of each ^18^F-labeled entity in the respective plasma TACs are known (18). These values were retrieved from the literature, specifically from Huang et al. (19), who used a hematocrit of 40%, and from Melega et al. (33), who reported the metabolite proportions for patients pre-medicated with 100 mg of Carbidopa, which is identical to the pre-medication schedule of our patients. To extrapolate the proportions of metabolites at any time, the measured fractions of plasma radioactivity were fitted using the following equations for the plasma fractions of DOPA, OMFD, and METS, respectively:


$$f_{DOPA}{\left(t\right)}_{p}=1-0.36902735\times (2-e^{-0.03915133t}-e^{-0.03915214t})$$

$$f_{OMFD}{\left(t\right)}_{p}=0.24080881\;\times (1-e^{-0.03251229t})+0.43768904\times (1-e^{-0.03251228t})$$

$$f_{METS}{\left(t\right)}_{p}=1-f_{OMFD}{\left(t\right)}_{p}-f_{DOPA}{\left(t\right)}_{p}$$

where *t* is the time in minutes.

### Dynamic Models

**Figure 1:**
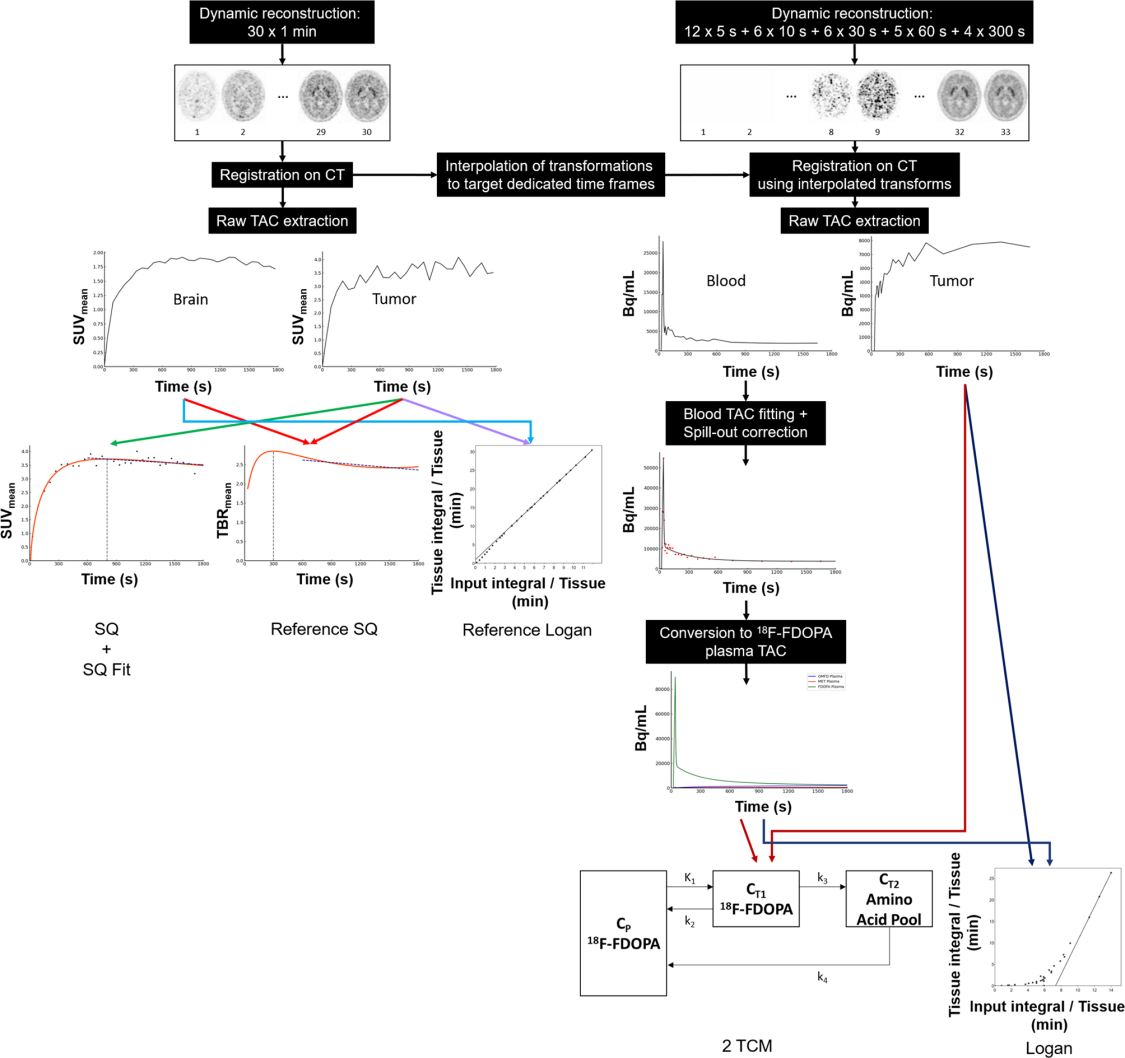
Workflow showing the pre-processing steps required to perform dynamic analyses for all of the dynamic models used in this study: semiquantitative model (*SQ*) semiquantitative fitting parameters (*SQ Fit*), reference semiquantitative model (*Reference SQ*), Logan model with input function (*Logan*), Logan model with reference region (*Reference Logan*), and the two-tissue compartmental model (2TCM). *TBR*, tumor-to-brain ratio.

The pre-processing steps and input data for each dynamic model are presented in **Figure 1**.

#### Semiquantitative Models

To overcome noise effects, tumor TACs were first fitted using nonlinear least square optimizations and a specific tumor vascularization function (patent WO/2008/053268, entitled “Method and System for Quantification of Tumoral Vascularization”) (8, 24, 34):


$$SUV(t)=\{0,\;t>t^{*}\;a_{0}+(a_{1}+a_{0})\left(\frac{A+{\left(\frac{\left(t-t^{*}\right)}{a_{2}}\right)}^{p}}{B+{\left(\frac{\left(t-t^{*}\right)}{a_{2}}\right)}^{q}}\right),\;t\geq t^{*}$$

where t^*^ is the time interval from injection to the arrival of the radiotracer and *a*_0_ the baseline intensity that is fixed at 0. *a*_1_ and *a*_2_ reflect the maximal value of contrast agent uptake and the time to peak intensity, respectively. *p* and *q* are coefficients related to the increase in intensity and the decrease in intensity, respectively. *A* and *B* are arbitrary parameters.

Semiquantitative (SQ) model parameters, time-to-peak (TTP) and slope, were respectively computed as the time from the beginning of the dynamic acquisition to the maximum uptake value and the slope of the linear regression of the data between the 10th and the 30th minute (8). Semiquantitative fitting parameters (SQ Fit)—*a*_1_, *a*_2_, *p*, *q*, *A*, and *B*—extracted from the equation of the fit were also assessed. The reference semiquantitative (Ref SQ) model was conducted as an assessment of other studies where the tumor-to-normal brain ratio dynamic values were used to overcome the Carbidopa effect (8, 24, 34), even though such normalization was not needed in this study. To achieve this, healthy brain TACs were fitted similarly to tumor TACs, and TAC ratios (TAC_ratio_), representing the evolution of the ratios between tumor and brain fitted TACs, were computed. The TTP_ratio_ and slope_ratio_ were computed from the TAC_ratio_ similarly to the tumor TACs.

#### Graphical Models

Among all graphical models available, the Logan graphical model (35) with the computation of the equilibrium volume of distribution is particularly suited to ^18^F-FDOPA in gliomas since there is no evidence that ^18^F-FDOPA is trapped in tumors (17). The Logan graphical model (Logan) was performed with the slope computed between 15 and 30 min post-injection, as previously suggested (17). The equilibrium volumes of distribution (*V*_ed_) and Int_Logan_ were computed as the slope and the intercept of the graphical model, respectively. We also used the Logan graphical model with a healthy brain reference region (Ref Logan) similarly to the Patlak graphical model for ^18^F-FDOPA PET imaging of parkinsonian syndromes (36). Two parameters, with regression between 15 and 30 min, were extracted from the Ref Logan model, namely, the distribution volume ratio (DVR) and the relative residence time (RRT), computed respectively as the slope and the negative intercept.

#### Compartmental Model

The model used was a simplified two-tissue compartmental model (2TCM) adapted from the original publication of Huang et al. (19) and previously used for the compartmental analysis of ^18^F-FDOPA glioma imaging (18). Four rate constants (*K*_1_, *k*_2_, *k*_3_, and *k*_4_) and the blood volume fraction (*V*_b_) were estimated by fitting the 2TCM to tumor TACs. The net influx rate constant, *Ki*, was computed from the previously estimated four rate constants as *Ki* = (*K*_1_ × *k*_3_) / (*k*_2_ + *k*_3_).

### Statistical Analysis

Categorical variables are expressed as numbers and percentages and continuous variables as medians (first quartile to third quartile) because the variables did not follow a normal distribution. Intergroup comparisons were performed with the chi-squared test for categorical variables and the Mann–Whitney test for continuous variables.

For the overall comparison of the different kinetic models, parameters belonging to the same model were used to construct a multivariate model. This multivariate model was a general linear model with variables selected automatically in a stepwise manner with both forward and backward selections minimizing the Akaike information criterion. Comparisons between the performances of the final models were carried out with the one-sided comparison of superiority pairwise Delong tests (37).

The ability of each individually extracted parameter to predict an IDH mutation was assessed using receiver operating characteristic (ROC) curves from which the area under the curve (AUC), sensitivity, specificity, and accuracy were computed. The optimal threshold was determined by selecting the point on the curve closest to (0,1). Spearman’s coefficients were calculated to assess the correlations between each extracted parameter of the different models.

*P*-values were adjusted using the Benjamini–Hochberg correction, and *p*-values lower than 0.05 were considered significant. Analyses were performed with the R software version 3.6.2 (R Foundation for Statistical Computing, Vienna, Austria).

## Results

### Patients

Forty-five patients were initially selected, but eight patients should have been subsequently excluded because of issues in input function determination. The final population thus consisted of 37 patients [median age = 58.3 years, range = 20.3–79.9 years, 16 (43%) women]. According to the WHO 2016 classification of gliomas, 8 gliomas (22%) were classified as IDH-mutant astrocytomas (one was an anaplastic glioma), 7 (19%) as IDH-wild-type astrocytomas (four were anaplastic gliomas), 5 (14%) as IDH-mutant and 1p/19q co-deleted oligodendrogliomas, 16 (43%) as IDH-wild-type glioblastomas, and 1 (3%) as IDH-mutant glioblastoma.

### Dynamic Models

**Figure 2:**
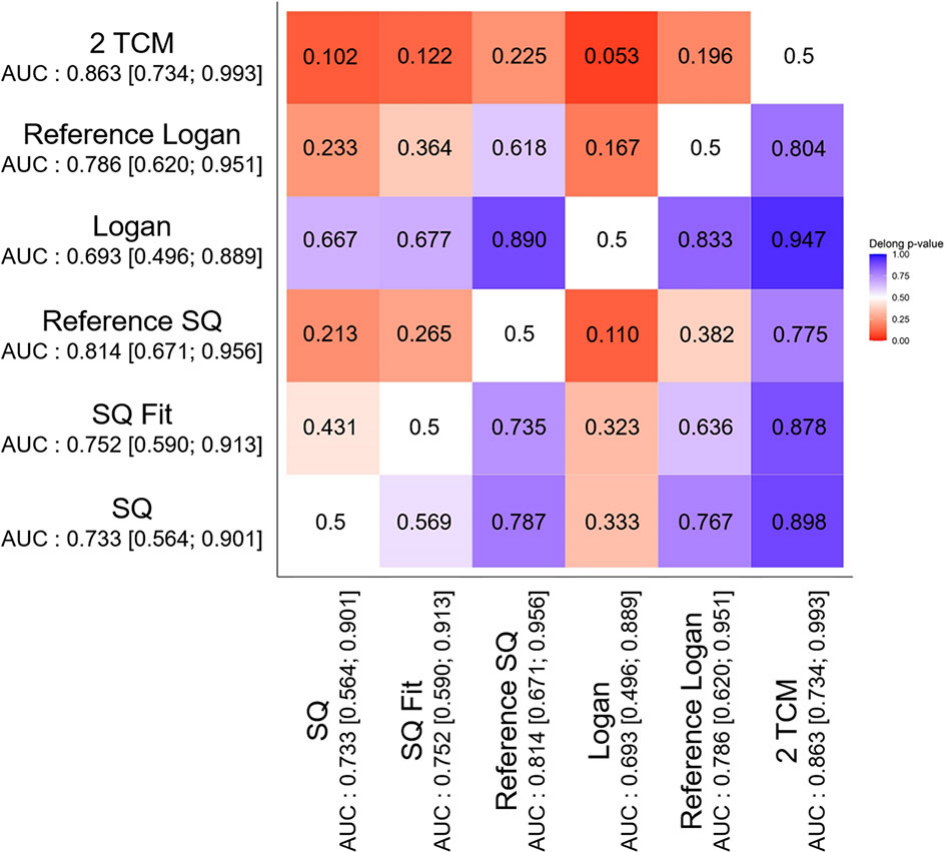
Heatmap of the *p*-values for one-sided comparison of superiority pairwise Delong tests of the AUC multivariate analyses performed for each dynamic model. *AUC*, area under the curve; *SQ*, semiquantitative model; *SQ Fit*, semiquantitative fitting parameters; *Reference SQ*, reference semiquantitative model; *2TCM*, two-tissue compartmental model.

The SQ model with an AUC of 0.733 showed similar performance to the other models with AUCs of 0.752, 0.814, 0.693, 0.786, and 0.863, respectively corresponding to SQ Fit, Ref SQ, Logan, Ref Logan, and 2TCM (*p* ≥ 0.10 for the pairwise comparisons with the other models). We tested all the possible pairwise dynamic model combinations and found no significant differences (*p* > 0.05) (**Table 1** and **Figure 2**).

**Table 1:** Results of the multivariate analyses for predicting isocitrate dehydrogenase (IDH) mutations with different dynamic models.

Dynamic model	AUC (95%CI)	Parameters included in the multivariate model
Semiquantitative	0.733 (0.564–0.901)	TTP
Semiquantitative fit	0.752 (0.590–0.913)	*p*
		*a*_2_
		*A*
Reference semiquantitative	0.814 (0.671–0.956)	TTP_ratio_
Logan	0.693 (0.496–0.889)	Int_Logan_
Reference Logan	0.786 (0.620–0.951)	DVR
		RRT
Two-tissue compartmental	0.866 (0.737–0.996)	*K*_1_
		*k*_3_
		*k*_4_

AUC, area under the curve; TTP, time-to-peak; DVR, distribution volume ratio; RRT, relative residence time.

**Figure 3:**
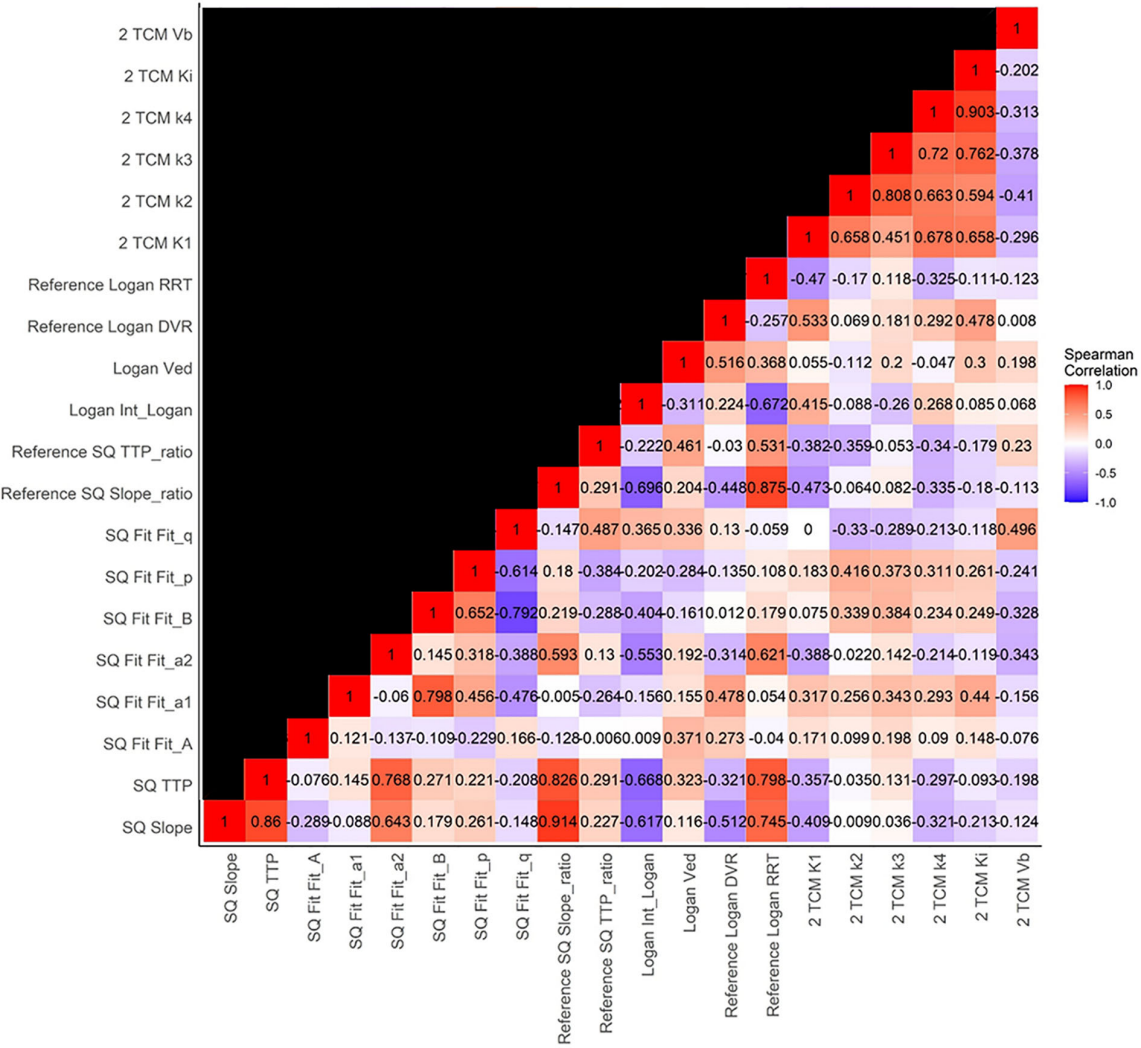
Heatmap of Spearman’s correlation coefficients for the individual parameters of each of the models.

**Figure 4:**
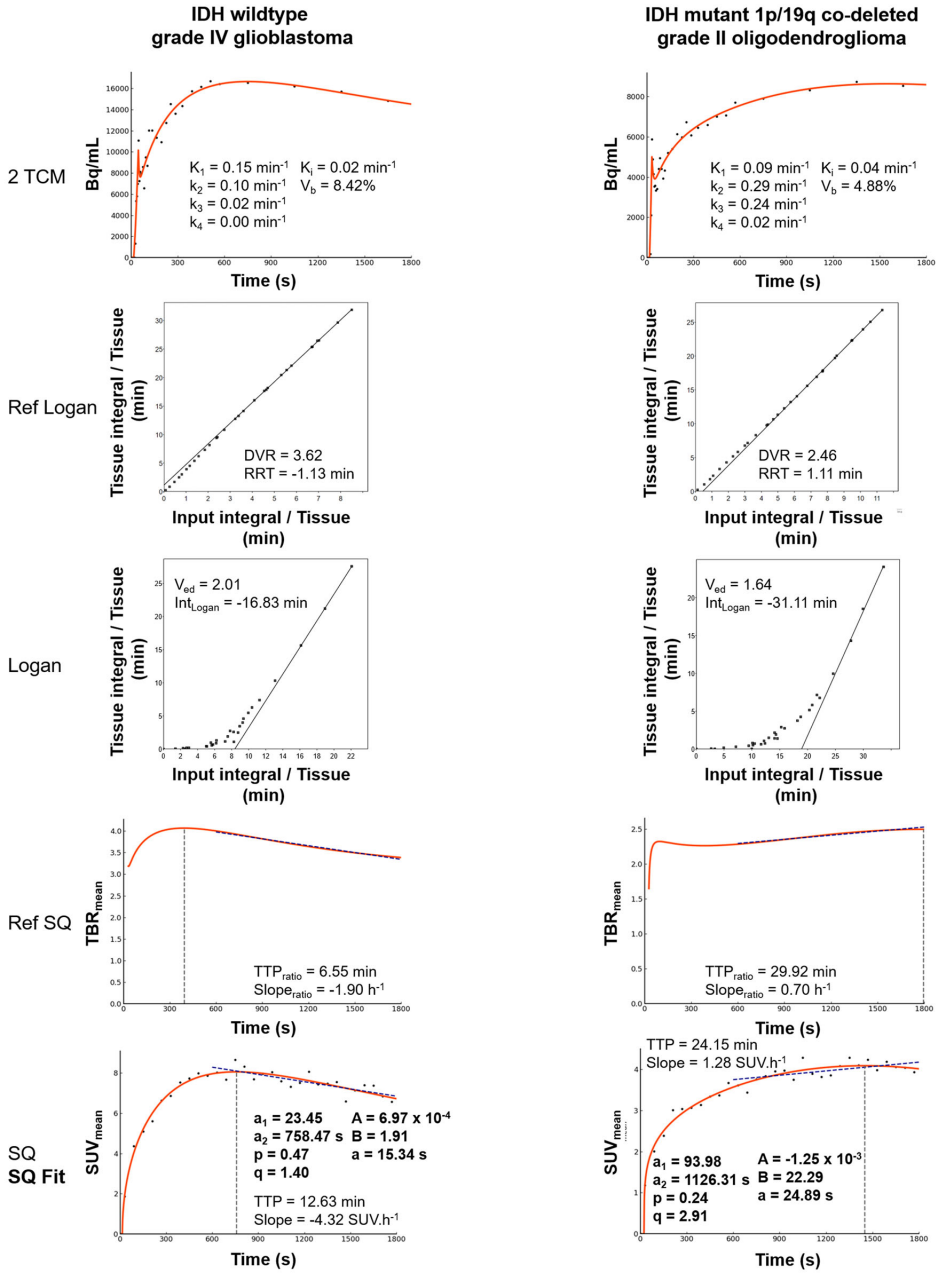
Representative IDH-wild-type grade IV glioblastoma and an IDH-mutant 1p/19q co-deleted grade II oligodendroglioma. Fitted results and parameters extracted from all the dynamic models used in this study are displayed for each of the two patients: semiquantitative model (*SQ*), semiquantitative fitting parameters (*SQ Fit*), reference semiquantitative model (*Reference SQ*), Logan model with input function (*Logan*), Logan model with reference region (*Reference Logan*), and the two-tissue compartmental model (2TCM). *IDH*, isocitrate dehydrogenase; *TBR*, tumor-to-brain ratio.

Detailed diagnostic performances of all individual parameters and the correlations between individual parameters are presented in **Table 2** and **Figure 3**, respectively. The TTP parameter from the SQ model showed the highest accuracy (75.7%) among all the parameters examined. The other SQ parameter, i.e., slope, also had a strong diagnostic performance, with an accuracy of 73.0%, which is within the range of performances for the parameters from the Ref SQ and Ref Logan models (respective accuracies of 73.0% and 70.3% for both TTP_ratio_ and Slope_ratio_ and both DVR and RRT). Despite its high overall performance, none of the parameters obtained with the 2TCM were significant for predicting IDH mutations (*p* > 0.14). SQ parameters were highly correlated with Ref SQ Slope_ratio_, the *a*_2_ parameter from SQ Fit, and the intercepts from Logan and Ref Logan, namely, Int_Logan_ and RRT, respectively. The 2TCM parameters, however, did not correlate with any of the parameters from the other models. Most parameters extracted from the 2TCM were highly intra-correlated. Representative IDH-wild-type and IDH-mutant gliomas with all the models considered are shown in **Figure 4**.

**Table 2:** Results of the receiver operating characteristic (ROC) curve analyses for predicting isocitrate dehydrogenase (IDH) mutations using individual parameters.

Dynamic model	Parameter	*p*-value	AUC	Cutoff	Sensitivity (%)	Specificity (%)	Accuracy (%)
Semiquantitative	TTP	**0.020**	0.733	18.41 min	57.1	87.0	75.7
	Slope	**0.020**	0.730	−0.55 SUV h^−1^	64.3	78.3	73.0
Semiquantitative fit	*a*_1_	0.393	0.630	14.67	57.1	73.9	67.6
	*a*_2_	0.390	0.652	936.86 s	71.4	60.9	64.9
	*p*	0.432	0.599	1.665	64.3	47.8	54.1
	*q*	0.390	0.668	0.299	71.4	65.2	67.6
	*A*	0.699	0.540	0	64.3	56.5	59.5
	*B*	0.432	0.593	2.21	50.0	78.3	67.6
Reference semiquantitative	TTP_ratio_	**0.003**	0.814	5.28 min	78.6	69.6	73.0
	Slope_ratio_	**0.024**	0.724	−0.37 h^−1^	78.6	69.6	73.0
Logan	*V*_ed_	0.147	0.646	1.51	64.3	69.6	67.6
	Int_Logan_	0.107	0.693	−24.85 min	57.1	82.6	73.0
Reference Logan	DVR	**0.046**	0.699	2.41	78.6	65.2	70.3
	RRT	**0.030**	0.739	−0.71 min	78.6	65.2	70.3
Two-tissue compartmental	*K*_1_	0.083	0.742	0.13 min^−1^	71.4	69.6	70.3
	*k*_2_	0.405	0.609	0.40 min^−1^	64.3	52.2	56.8
	*k*_3_	0.888	0.516	0.18 min^−1^	57.1	56.5	56.8
	*k*_4_	0.257	0.671	0.03 min^−1^	71.4	65.2	67.6
	*Ki*	0.405	0.599	0.03 min^−1^	64.3	56.5	59.5
	*V*_b_	0.405	0.596	1.97	71.4	56.5	62.2

Bold p-values are significant Mann–Whitney tests for the comparison of IDH-wild-type and IDH-mutant gliomas.

AUC, area under the curve; SUV, standard uptake value; TTP, time-to-peak; DVR, distribution volume ratio; RRT, relative residence time.

## Discussion

The current study compared different ^18^F-FDOPA kinetic models to assess whether the SQ model, which is currently recommended for amino acid PET in clinical routine practice, is specifically suited to a ^18^F-FDOPA PET dynamic analysis (12). This comparison was performed on newly diagnosed gliomas for which the amino acid PET dynamic analysis had previously been shown to successfully predict the status of IDH mutation (6, 8, 24). The SQ model was found to have a similar performance to the other models, including the 2TCM, as previously reported for the dynamic ^18^F-FDOPA PET imaging of gliomas (17, 18) (**Table 1** and **Figure 2**). Our study therefore provides supportive evidence for the application of the SQ model, to predict IDH mutations by ^18^F-FDOPA PET imaging in newly diagnosed gliomas, in the clinic.

The SQ model has the advantage of being easily integrated into clinical routine practice with no need for heavy processing, a reference region, or an input function. Although compartmental analysis, which is considered the current gold standard, requires an input function, input functions are notoriously difficult to obtain in clinical routine practice because they either require blood sampling or, if extracted directly from images, heavy post-processing and many approximations (see **Figure 1**), which limits their use in the clinic.

There has been renewed interest in dynamic PET acquisitions because of the significant technological advances in the field, such as digital signal (38) and/or the longer field of view of PET devices. These improvements make dynamic acquisitions accessible to clinical routine practice (39). A simple dynamic model is thus preferable to harmonize protocols across different centers and to promote a wider acceptance in the scientific medical community, not only among nuclear physicians but also clinicians requiring access to easily understandable and interpretable data.

Our study confirms the high diagnostic performance of dynamic ^18^F-FDOPA PET imaging using the SQ model to predict IDH mutations, with the TTP analysis yielding an accuracy of 75.7%. TACs with shorter TTPs may, in addition, identify more aggressive IDH-wild-type gliomas, which may correspond not only to tumors that express high concentrations of LAT transporters (40) but also those that are characterized by more extensive tracer perfusion, as shown for ^18^F-FET (11) and discussed in our previous publications (8, 24). Such performances are within the range of previously published studies that reported accuracies of 74% for TTP alone (8) and 75% when associated with other specific radiomics features of ^18^F-FDOPA PET imaging (24). It should nevertheless be noted that these latter two studies used a reference region for the SQ model to account for the population heterogeneity with regard to the Carbidopa pre-medication (8, 24, 34). In the current study, we report similar overall performances of the Ref SQ model and the SQ model (Ref SQ: AUC = 0.814, 95% CI = 0.671–0.956, *p* = 0.21, for the pairwise test with the SQ model). This is consistent with results from our previous dynamic ^18^F-FDOPA PET studies (8, 24, 34). Even though no significant differences were observed between the different models studied, the 2TCM provided better performance (AUC = 0.863, range = 0.734–0.993, *p* = 0.11, for the pairwise test with the SQ model) with parameters that reflect different kinetic information compared to those from the other models (**Figure 3**). However, as explained above, adapting the 2TCM to clinical routine practice presents serious challenges and would make it more difficult to harmonize data sourced from different centers.

Among the radiolabeled amino acids recommended for the assessment of gliomas, ^18^F-FET and ^18^F-FDOPA have been demonstrated to have similar semiquantitative static parameters (41) but different kinetic parameters for tumor grading using compartmental modeling (28). When applying the WHO 2016 classification of gliomas, dynamic parameters for both radiotracers extracted from the SQ or the Ref SQ model, in the literature, showed similar performances in predicting IDH mutations. An accuracy of 72% was reported for the ^18^F-FET TTP (6), while an accuracy of 74% was described for the ^18^F-FDOPA TTP_ratio_ (8). These results were also confirmed in the Lohmann et al. radiomics study, which reported a model accuracy of 80% (42). The study of Lohmann et al. nevertheless showed that dynamic parameters were particularly pertinent in determining diagnostic performance. Since the underlying kinetic model of ^18^F-FDOPA is much more complex than that of ^18^F-FET, there was no evidence that a simple model such as the SQ model would suffice. Our current study showed that the SQ model is appropriate for interpreting ^18^F-FDOPA kinetics and that it might be considered in future guidelines, as is currently the case for ^18^F-FET imaging (12).

The main limitations of our study are related to the assumptions that underpin how the input function was determined. Since no blood sampling was performed for any of our patients, we extrapolated the input function from an image-derived blood TAC that was converted to a ^18^F-FDOPA plasma TAC using a previously published method (18). This method is based on hematocrit data and the proportions of individual metabolites previously reported in the literature (19, 33). Since this assumption is not adapted to each patient, it may have a negative impact on the results from the model that are dependent on this input function. Moreover, our study used a digital PET device with high count sensitivity and improved dynamic image quality. The values of the different parameters may therefore not be directly comparable to those extracted from noisier dynamic images, captured with older PET devices. This may significantly affect the fitting process that is used for each dynamic model and, thus, affect the stability and the ability to directly extrapolate our results to other PET devices. The number of patients in this study was also limited, even though the diagnostic performances reported were similar to those of other amino acid dynamic PET studies. Although our population comprised a highly selected group of patients, it included representative proportions of the different types of gliomas (43).

## Conclusion

Despite the complexities of the ^18^F-FDOPA kinetic model, our study confirms that a simple semiquantitative analysis, which is currently recommended for amino acid PET imaging in clinical routine practice, captures all the relevant information contained in TACs to predict the presence of IDH mutations when compared to the more sophisticated graphical and compartmental models. Although dynamic parameters play important roles in the interpretation of radiolabeled amino acid PET imaging in gliomas, our current study shows that the application of this easily transposable method can be extended to ^18^F-FDOPA PET imaging for the noninvasive characterization of newly diagnosed gliomas.

## Data Availability Statement

The original contributions presented in the study are included in the article/**Supplementary Material**. Further inquiries can be directed to the corresponding author.

## Ethics Statement

The studies involving human participants were reviewed and approved by Comité d’Ethique du CHRU de Nancy - FRANCE. The patients/participants provided their written informed consent to participate in this study.

## Author Contributions

TZ, MD, and AV conceived and designed the study and analyzed the data. FR, MB, LT, and LI participated either in the study conception and design or the analysis and interpretation of the data. All authors helped with the drafting of the manuscript or revising it critically for important intellectual content. FR, MB, LT, LI, and AV approved the submitted version. All authors contributed to the article and approved the submitted version.

## Conflict of Interest

The authors declare that the research was conducted in the absence of any commercial or financial relationships that could be construed as a potential conflict of interest.

## Publisher’s Note

All claims expressed in this article are solely those of the authors and do not necessarily represent those of their affiliated organizations, or those of the publisher, the editors and the reviewers. Any product that may be evaluated in this article, or claim that may be made by its manufacturer, is not guaranteed or endorsed by the publisher.

## Supplementary Material

Supplementary Table 1 Raw_TACs_30x60s.

Supplementary Table 2 Raw_TACs_compart_temp_sampling.

Supplementary Table 3 Clinical_data.

Supplementary Table 4 Parameters_results.

## References

[1] Albert NL Weller M Suchorska B Galldiks N Soffietti R Kim MM. Response Assessment in Neuro-Oncology Working Group and European Association for Neuro-Oncology Recommendations for the Clinical Use of PET Imaging in Gliomas. Neuro-Oncol (2016) 18:1199–208. doi: 10.1093/neuonc/now058 PMC4999003 27106405

[2] Vettermann F Suchorska B Unterrainer M Nelwan D Forbrig R Ruf V. Non-Invasive Prediction of IDH-Wildtype Genotype in Gliomas Using Dynamic 18F-FET PET. Eur J Nucl Med Mol Imaging (2019) 46:2581–9. doi: 10.1007/s00259-019-04477-3 31410540

[3] Kunz M Albert NL Unterrainer M la Fougere C Egensperger R Schüller U. Dynamic 18f-FET PET Is a Powerful Imaging Biomarker in Gadolinium-Negative Gliomas. Neuro-Oncol (2019) 21:274–84. doi: 10.1093/neuonc/noy098 PMC6374762 29893965

[4] Jansen NL Graute V Armbruster L Suchorska B Lutz J Eigenbrod S. MRI-Suspected Low-Grade Glioma: Is There a Need to Perform Dynamic FET PET? Eur J Nucl Med Mol Imaging (2012) 39:1021–9. doi: 10.1007/s00259-012-2109-9 22491781

[5] Obara T Blonski M Brzenczek C Mézières S Gaudeau Y Pouget C. Adult Diffuse Low-Grade Gliomas: 35-Year Experience at the Nancy France Neurooncology Unit. Front Oncol (2020) 10:574679. doi: 10.3389/fonc.2020.574679 33194684 PMC7656991

[6] Verger A Stoffels G Bauer EK Lohmann P Blau T Fink GR. Static and Dynamic 18F–FET PET for the Characterization of Gliomas Defined by IDH and 1p/19q Status. Eur J Nucl Med Mol Imaging (2018) 45:443–51. doi: 10.1007/s00259-017-3846-6 29043400

[7] Lopci E Riva M Olivari L Raneri F Soffietti R Piccardo A. Prognostic Value of Molecular and Imaging Biomarkers in Patients With Supratentorial Glioma. Eur J Nucl Med Mol Imaging (2017) 44:1155–64. doi: 10.1007/s00259-017-3618-3 28110346

[8] Ginet M Zaragori T Marie P-Y Roch V Gauchotte G Rech F. Integration of Dynamic Parameters in the Analysis of 18F-FDopa PET Imaging Improves the Prediction of Molecular Features of Gliomas. Eur J Nucl Med Mol Imaging (2020) 47:1381–90. doi: 10.1007/s00259-019-04509-y 31529264

[9] Nomura Y Asano Y Shinoda J Yano H Ikegame Y Kawasaki T. Characteristics of Time-Activity Curves Obtained From Dynamic 11C-Methionine PET in Common Primary Brain Tumors. J Neurooncol (2018) 138:649–58. doi: 10.1007/s11060-018-2834-4 29564749

[10] Louis DN Perry A Wesseling P Brat DJ Cree IA Figarella-Branger D. The 2021 WHO Classification of Tumors of the Central Nervous System: A Summary. Neuro-Oncol (2021) 23(8):1231–51. doi: 10.1093/neuonc/noab106 PMC8328013 34185076

[11] Pöpperl G Kreth FW Herms J Koch W Mehrkens JH Gildehaus FJ. Analysis of 18F-FET PET for Grading of Recurrent Gliomas: Is Evaluation of Uptake Kinetics Superior to Standard Methods? J Nucl Med (2006) 47:393–403. 16513607

[12] Law I Albert NL Arbizu J Boellaard R Drzezga A Galldiks N. Joint EANM/EANO/RANO Practice Guidelines/SNMMI Procedure Standards for Imaging of Gliomas Using PET With Radiolabelled Amino Acids and [18F]FDG: Version 1.0. Eur J Nucl Med Mol Imaging (2019) 46:540–57. doi: 10.1007/s00259-018-4207-9 PMC6351513 30519867

[13] Wester HJ Herz M Weber W Heiss P Senekowitsch-Schmidtke R Schwaiger M. Synthesis and Radiopharmacology of O-(2-[18F]Fluoroethyl)-L-Tyrosine for Tumor Imaging. J Nucl Med (1999) 40:205–12. 9935078

[14] Debus C Afshar-Oromieh A Floca R Ingrisch M Knoll M Debus J. Feasibility and Robustness of Dynamic 18F-FET PET Based Tracer Kinetic Models Applied to Patients With Recurrent High-Grade Glioma Prior to Carbon Ion Irradiation. Sci Rep (2018) 8:14760. doi: 10.1038/s41598-018-33034-5 30283013 PMC6170489

[15] Bolcaen J Lybaert K Moerman L Descamps B Deblaere K Boterberg T. Kinetic Modeling and Graphical Analysis of 18F-Fluoromethylcholine (FCho), 18f-Fluoroethyltyrosine (FET) and 18F-Fluorodeoxyglucose (FDG) PET for the Fiscrimination Between High-Grade Glioma and Radiation Necrosis in Rats. PloS One (2016) 11:e0161845. doi: 10.1371/journal.pone.0161845 27559736 PMC4999092

[16] Thiele F Ehmer J Piroth MD Eble MJ Coenen HH Kaiser H-J. The Quantification of Dynamic FET PET Imaging and Correlation With the Clinical Outcome in Patients With Glioblastoma. Phys Med Biol (2009) 54:5525–39. doi: 10.1088/0031-9155/54/18/012 19717889

[17] Schiepers C Chen W Cloughesy T Dahlbom M Huang S-C. 18f-FDOPA Kinetics in Brain Tumors. J Nucl Med (2007) 48:1651–61. doi: 10.2967/jnumed.106.039321 17873130

[18] Wardak M Schiepers C Cloughesy TF Dahlbom M Phelps ME Huang S-C. 18f-FLT and 18F-FDOPA PET Kinetics in Recurrent Brain Tumors. Eur J Nucl Med Mol Imaging (2014) 41:1199–209. doi: 10.1007/s00259-013-2678-2 PMC4008691 24604590

[19] Huang S-C Yu D-C Barrio JR Grafton S Melega WP Hoffman JM. Kinetics and Modeling of L-6-[^18^ F]Fluoro-DOPA in Human Positron Emission Tomographic Studies. J Cereb Blood Flow Metab (1991) 11:898–913. doi: 10.1038/jcbfm.1991.155 1939385

[20] Louis DN Perry A Reifenberger G von Deimling A Figarella-Branger D Cavenee WK. The 2016 World Health Organization Classification of Tumors of the Central Nervous System: A Summary. Acta Neuropathol (Berl) (2016) 131:803–20. doi: 10.1007/s00401-016-1545-1 27157931

[21] Galldiks N Unterrainer M Judov N Stoffels G Rapp M Lohmann P. Photopenic Defects on O-(2-[18F]-Fluoroethyl)-L-Tyrosine PET: Clinical Relevance in Glioma Patients. Neuro-Oncol (2019) 21:1331–8. doi: 10.1093/neuonc/noz083 PMC6784268 31077276

[22] Zaragori T Castello A Guedj E Girard A Galldiks N Albert NL. Photopenic Defects in Gliomas With Amino-Acid PET and Relative Prognostic Value: A Multicentric 11c-Methionine and 18F-FDOPA PET Experience. Clin Nucl Med (2021) 46:e36–7. doi: 10.1097/RLU.0000000000003240 32804767

[23] Janvier L Olivier P Blonski M Morel O Vignaud J-M Karcher G. Correlation of SUV-Derived Indices With Tumoral Aggressiveness of Gliomas in Static 18f-FDOPA PET: Use in Clinical Practice. Clin Nucl Med (2015) 40:e429–35. doi: 10.1097/RLU.0000000000000897 26204212

[24] Zaragori T Oster J Roch V Hossu G Chawki MB Grignon R. ^18^ F-FDOPA PET for the Non-Invasive Prediction of Glioma Molecular Parameters: A Radiomics Study. J Nucl Med (2021) jnumed.120.261545. doi: 10.2967/jnumed.120.261545 PMC8717204 34016731

[25] Salvadori J Imbert L Perrin M Karcher G Lamiral Z Marie P-Y. Head-To-Head Comparison of Image Quality Between Brain 18F-FDG Images Recorded With a Fully Digital Versus a Last-Generation Analog PET Camera. EJNMMI Res (2019) 9:61. doi: 10.1186/s13550-019-0526-5 31300962 PMC6626093

[26] Unterrainer M Vettermann F Brendel M Holzgreve A Lifschitz M Zähringer M. Towards Standardization of 18F-FET PET Imaging: Do We Need a Consistent Method of Background Activity Assessment? EJNMMI Res (2017) 7:48. doi: 10.1186/s13550-017-0295-y 28560582 PMC5449315

[27] Cicone F Carideo L Minniti G Scopinaro F. The Mean Striatal 18F-DOPA Uptake Is Not a Reliable Cut-Off Threshold for Biological Tumour Volume Definition of Glioma. Eur J Nucl Med Mol Imaging (2019) 46:1051–3. doi: 10.1007/s00259-019-4276-4 30685796

[28] Kratochwil C Combs SE Leotta K Afshar-Oromieh A Rieken S Debus J. Intra-Individual Comparison of 18F-FET and 18F-DOPA in PET Imaging of Recurrent Brain Tumors. Neuro-Oncol (2014) 16:434–40. doi: 10.1093/neuonc/not199 PMC3922512 24305717

[29] Nioche C Orlhac F Boughdad S Reuzé S Goya-Outi J Robert C. LIFEx: A Freeware for Radiomic Feature Calculation in Multimodality Imaging to Accelerate Advances in the Characterization of Tumor Heterogeneity. Cancer Res (2018) 78:4786–9. doi: 10.1158/0008-5472.CAN-18-0125 29959149

[30] Ye H Wong K-P Wardak M Dahlbom M Kepe V Barrio JR. Automated Movement Correction for Dynamic PET/CT Images: Evaluation With Phantom and Patient Data. PloS One (2014) 9:e103745. doi: 10.1371/journal.pone.0103745 25111700 PMC4128781

[31] Zanotti-Fregonara P Hirvonen J Lyoo CH Zoghbi SS Rallis-Frutos D Huestis MA. Population-Based Input Function Modeling for [18F]FMPEP-D2, an Inverse Agonist Radioligand for Cannabinoid CB1 Receptors: Validation in Clinical Studies. PloS One (2013) 8:e60231. doi: 10.1371/journal.pone.0060231 23577094 PMC3618181

[32] Martens C Debeir O Decaestecker C Metens T Lebrun L Leurquin-Sterk G. Voxelwise Principal Component Analysis of Dynamic [S-Methyl-11c]Methionine PET Data in Glioma Patients. Cancers (2021) 13:2342. doi: 10.3390/cancers13102342 34066294 PMC8152079

[33] Melega WP Grafton ST Huang S-C Satyamurthy N Phelps ME Barrio JR. L-6-[^18^ F]Fluoro-DOPA Metabolism in Monkeys and Humans: Biochemical Parameters for the Formulation of Tracer Kinetic Models With Positron Emission Tomography. J Cereb Blood Flow Metab (1991) 11:890–7. doi: 10.1038/jcbfm.1991.154 1939384

[34] Zaragori T Ginet M Marie P-Y Roch V Grignon R Gauchotte G. Use of Static and Dynamic [^18^F]-F-DOPA PET Parameters for Detecting Patients With Glioma Recurrence or Progression. EJNMMI Res (2020) 10:1–10. doi: 10.1186/s13550-020-00645-x 32472232 PMC7260331

[35] Logan J. Graphical Analysis of PET Data Applied to Reversible and Irreversible Tracers. Nucl Med Biol (2000) 27:661–70. doi: 10.1016/s0969-8051(00)00137-2 11091109

[36] Morbelli S Esposito G Arbizu J Barthel H Boellaard R Bohnen NI. EANM Practice Guideline/SNMMI Procedure Standard for Dopaminergic Imaging in Parkinsonian Syndromes 1.0. Eur J Nucl Med Mol Imaging (2020) 47:1885–912. doi: 10.1007/s00259-020-04817-8 PMC7300075 32388612

[37] DeLong ER DeLong DM Clarke-Pearson DL. Comparing the Areas Under Two or More Correlated Receiver Operating Characteristic Curves: A Nonparametric Approach. Biometrics (1988) 44:837. doi: 10.2307/2531595 3203132

[38] Salvadori J Odille F Verger A Olivier P Karcher G Marie P-Y. Head-To-Head Comparison Between Digital and Analog PET of Human and Phantom Images When Optimized for Maximizing the Signal-to-Noise Ratio From Small Lesions. EJNMMI Phys (2020) 7:11. doi: 10.1186/s40658-020-0281-8 32086646 PMC7035408

[39] Dimitrakopoulou-Strauss A Pan L Sachpekidis C. Kinetic Modeling and Parametric Imaging With Dynamic PET for Oncological Applications: General Considerations, Current Clinical Applications, and Future Perspectives. Eur J Nucl Med Mol Imaging (2020) 48:21–39. doi: 10.1007/s00259-020-04843-6 32430580 PMC7835173

[40] Dadone-Montaudié B Ambrosetti D Dufour M Darcourt J Almairac F Coyne J. [18f] FDOPA Standardized Uptake Values of Brain Tumors Are Not Exclusively Dependent on LAT1 Expression. PloS One (2017) 12:e0184625. doi: 10.1371/journal.pone.0184625 28937983 PMC5609741

[41] Lapa C Linsenmann T Monoranu CM Samnick S Buck AK Bluemel C. Comparison of the Amino Acid Tracers 18F-FET and 18F-DOPA in High-Grade Glioma Patients. J Nucl Med (2014) 55:1611–6. doi: 10.2967/jnumed.114.140608 25125481

[42] Lohmann P Lerche C Bauer EK Steger J Stoffels G Blau T. Predicting IDH Genotype in Gliomas Using FET PET Radiomics. Sci Rep (2018) 8:13328. doi: 10.1038/s41598-018-31806-7 30190592 PMC6127131

[43] Jiang H Cui Y Wang J Lin S. Impact of Epidemiological Characteristics of Supratentorial Gliomas in Adults Brought About by the 2016 World Health Organization Classification of Tumors of the Central Nervous System. Oncotarget (2017) 131:803–20. doi: 10.18632/oncotarget.13555 PMC5386767 27888628

